# The complete mitochondrial genome of *Tibetan hulless* barley

**DOI:** 10.1080/23802359.2016.1180553

**Published:** 2016-07-06

**Authors:** Yu lin Wang, Ze Xiu Wei, Qi Jun Xu, Xing Quan Zeng, Hong Jun Yuan, Ya Wei Tang, Nyima Tashi

**Affiliations:** a Tibet Academy of Agricultural and Animal Husbandry Sciences, Lhasa, Tibet, China;; b Barley Improvement and Yak Breeding Key Laboratory, Lhasa, Tibet, China;; c Agricultural Resources and Environment Institute, Tibet Academy of Agricultural and Animal Husbandry Sciences, Lhasa, Tibet, China;; d Agricultural Research Institute, Tibet Academy of Agricultural and Animal Husbandry Sciences, Lhasa, Tibet, China

**Keywords:** *Hordeum vulgar* L. var. *nudum*, hulless barley, mitochondrial genome

## Abstract

Hulless barley (*Hordeum vulgar* L. var. *nudum*) is one of the staple foods for Tibetans and an important livestock feed in the Tibetan Plateau. We report the complete mitochondrial genome of *Tibetan hulless* barely. The complete mitochondrial genome size is 416,675 bp. Hulless barely mitochondrial genome encode 34 protein-coding genes, 19 tRNA genes and three rRNA genes. The mitochondrial genome has 50 forward and palindrome repeats totally. Nucleotide sequence of coding region takes over 13.60%, GC content is 44.33%. The maximum-likelihood (ML) phylogenetic tree based on 11 protein-coding genes common to seven plant mitochondrial genomes using *Arabidopsis thaliana* as out-group support that hulless barely is close to *Triticum* species.

Mitochondrial DNA is the extra-nuclear genome in plant cytoplasm except the chloroplast DNA, most likely through the taming of an α-proteobacterial endosymbiont. Lots of evidences support that a single origin of mitochondria for all eukaryotes originated about 1.5 billion years ago (Gupta & Golding 1996). Hulless barley as a main food crops and livestock feed more than important of rice where be cultivated in the Tibetan Plateau that high altitude and low temperature. Although the researchers had published the nuclear and chloroplast genome of hulless barely (Zeng et al. 2015a, 2015b), but mitochondrial genome of hulless barely remains unknown.

Fresh leaf of “Zangqing 320” was used to extract the mitochondrial genome DNA by CTAB protocol (Doyle 1987) that has been cultivated in the Tibet Academy of Agricultural and Animal Husbandry Sciences of China. Sequencing was performed on the Hiseq 2000 platform for short insert size libraries (PE500). First, Trimmomatic Version 0.22 (Bolger et al. 2014) and Perl scripts were used to data cleaning. Second, compared the clean reads with 75 mitochondrial genomes (ftp://ftp.ncbi.nlm.nih.gov/refseq/release/mitochondrion) by blastn (parameter settings: *E*-value = l*e*^−10^, the other parameters are defaults) (Altschul et al. 1990). Identity ≥98%, and the ratio of the reads length greater than or equal to 90% was defined as the mitochondrial reads. Thirdly, the mitochondrial reads were assembled by Platnus (Kajitani et al. 2014) and GapFiller (Boetzer & Pirovano 2012). Five random selected fragments (length=∼1 kB) were verified by PCR method. Finally, the mitochondrial genome was annotated by mitofy (Alverson et al. 2010). The GenBank accession number is KU865690.

Repetitive sequence was identified by REPuter (version v3.0, with a minimal length of 50 bp and three mismatches) (Kurtz et al. 2001). Reverse and complement type were not detected in hulless barely mitochondrial genome. The totally number of repeats are 50. Forward and palindrome repeats are 29 and 21, respectively. The longest forward and palindrome repeats are 7061 bp and 2366 bp, respectively.

The complete mitochondrial genome of hulless barley has a length of 416,675 bp with a typical circle structure that contains 19 tRNA genes, 34 protein-coding genes and three rRNA genes. Moreover, there are more than one copy of atp8, rRNA and tRNA genes making a total of 88 genes present in hulless barely mitochondrial genome. The percentage of coding region is 13.60%, and GC content is 44.33%.

We used 11 protein-coding genes (*atp1, atp9, cox1, cox2, cox3, nad4L, nad5, nad6, nad9, rps4* and *rps12*) common to seven plant mitochondrial genomes for our phylogenetic analysis. Totally 11 protein-coding genes were aligned using the MAFFT version 5 (Katoh et al. 2005). Maximum-likelihood analysis was implemented in RAxML version 8.2.4 (Parameters: -# 1000 -m GTRGAMMA –f a) (Stamatakis 2006). All other parameters are set as default (Figure 1).

**Figure 1.**
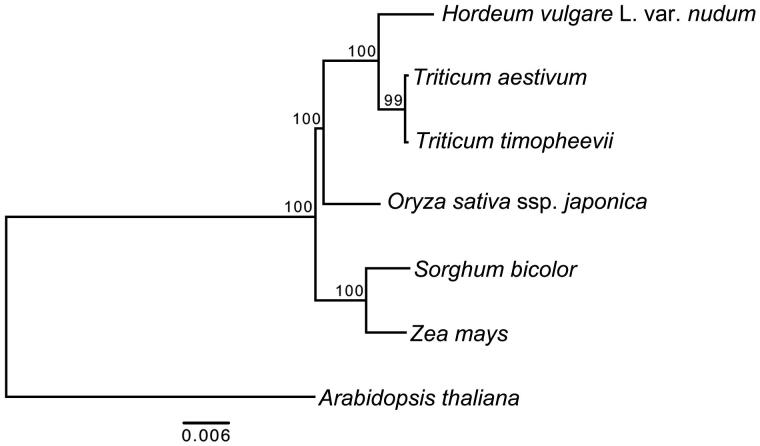
Maximum-likelihood phylogenetic tree based on 11 protein-coding genes common to seven plant mitochondrial genomes using *Arabidopsis thaliana* as an out-group. Accession numbers are as follows: *Arabidopsis thaliana* (NC_001284), *Oryza sativa* ssp. japonica (NC_011033), *Sorghum bicolor* (NC_008360), *Triticum aestivum* (NC_007579), *Triticum timopheevii* (NC_022714) and *Zea mays* (NC_007982).

Sequencing the mitochondrial genome of hulless barley will provide essential genomic information for breeding, together with its nuclear and chloroplast genomes is essence to understanding the diversification and evolution of hulless barely.

## References

[CIT0001] Altschul SF, Gish W, Miller W, Myers EW, Lipman DJ 1990 Basic local alignment search tool. J Mol Biol. 215:403–410. 2231712 10.1016/S0022-2836(05)80360-2

[CIT0002] Alverson AJ, Wei X, Rice DW, Stern DB, Barry K, Palmer JD 2010 Insights into the evolution of mitochondrial genome size from complete sequences of *Citrullus lanatus* and *Cucurbita pepo* (Cucurbitaceae). Mol Biol Evol. 27:1436–1448. 20118192 10.1093/molbev/msq029 PMC2877997

[CIT0003] Boetzer M, Pirovano W 2012 Toward almost closed genomes with GapFiller. Genome Biol. 13:R56 22731987 10.1186/gb-2012-13-6-r56 PMC3446322

[CIT0004] Bolger AM, Lohse M, Usadel B 2014 Trimmomatic: a flexible trimmer for Illumina sequence data. Bioinformatics. 30:2114–2120. 24695404 10.1093/bioinformatics/btu170 PMC4103590

[CIT0005] Doyle JJ 1987 A rapid DNA isolation procedure for small quantities of fresh leaf tissue. Phytochem Bull. 19:11–15.

[CIT0007] Gupta RS, Golding GB 1996 The origin of the eukaryotic cell. Trends Biochem Sci. 21:166–171. 8871398

[CIT0010] Kajitani R, Toshimoto K, Noguchi H, Toyoda A, Ogura Y, Okuno M, Yabana M, Harada M, Nagayasu E, Maruyama H, et al 2014 Efficient de novo assembly of highly heterozygous genomes from whole-genome shotgun short reads. Genome Res. 24:1384–1395. 24755901 10.1101/gr.170720.113 PMC4120091

[CIT0008] Katoh K, Kuma KI, Toh H, Miyata T 2005 MAFFT version 5: improvement in accuracy of multiple sequence alignment. Nucl Acids Res. 33:511–518. 15661851 10.1093/nar/gki198 PMC548345

[CIT0009] Kurtz S, Choudhuri JV, Ohlebusch E, Schleiermacher C, Stoye J, Giegerich R 2001 REPuter: the manifold applications of repeat analysis on a genomic scale. Nucleic Acids Res. 29:4633–4642. 11713313 10.1093/nar/29.22.4633 PMC92531

[CIT0011] Stamatakis A 2006 RAxML-VI-HPC: maximum likelihood-based phylogenetic analyses with thousands of taxa and mixed models. Bioinformatics. 22:2688–2690. 16928733 10.1093/bioinformatics/btl446

[CIT0012] Zeng X, Yuan J, Wang L, Xu J, Tashi N 2015a The complete chloroplast genome of Tibetan hulless barley. Mitochondrial DNA 29. [Epub ahead of print]. DOI: 10.3109/19401736.2015.1122765. 26713356

[CIT0013] Zeng X, Long H, Wang Z, Zhao S, Tang Y, Huang Z, Tashi N 2015b The draft genome of *Tibetan hulless* barley reveals adaptive patterns to the high stressful Tibetan Plateau. Proc Natl Acad Sci USA. 112:1095–1100. 25583503 10.1073/pnas.1423628112 PMC4313863

